# Recent advances of wide-angle metalenses: principle, design, and applications

**DOI:** 10.1515/nanoph-2021-0583

**Published:** 2021-12-02

**Authors:** XianGang Luo, Fei Zhang, MingBo Pu, YingHui Guo, Xiong Li, XiaoLiang Ma

**Affiliations:** State Key Laboratory of Optical Technologies on Nano-Fabrication and Micro-Engineering, Institute of Optics and Electronics Chinese Academy of Sciences, Chengdu 610209, China; School of Optoelectronics, University of Chinese Academy of Sciences, Beijing 100049, China

**Keywords:** flat optics, large field of view imaging, metasurfaces, wide-angle metalenses

## Abstract

Optical imaging systems, like microscopes, cameras, and telescopes, continue to expand the scope of human observation of the world. As one of the key indicators of imaging systems, the field-of-view (FOV) is often limited by coma aberration. Expanding it generally relies on a combination of complex lenses, leading to a bulky and cumbersome system. Recently, the emergency of meta-optics provides an alternative to constructing compact and lightweight large-FOV metalens through elaborated phase modulation within a flat surface, showing great potential in surveillance, unmanned vehicles, onboard planes or satellites, medical science, and other new applications. In this article, we review recent advances of wide-angle metalenses, including operation principles, design strategies, and application demos. Firstly, basic principles of wide-angle imaging using a single metalens are interpreted. Secondly, some advanced methods for designing subwavelength structures with high angle robustness and high efficiency are discussed. Thirdly, some representative functional devices and applications are surveyed. Finally, we conclude with an outlook on future potentials and challenges that need to be overcome.

## 1 Introduction

As an effective expansion of the human eye, optical imaging systems like microscopes and telescopes open the door to the micro world and the macro world, wherein classical laws of refraction and reflection have underpinned the development of optical systems for hundreds of years. Although traditional optics has achieved great success, it still suffers from some inherent problems such as large volume, heavy weight, and single function, posing an obstacle for the urgent requirement of modern optical systems for integrated, planar, and multifunctional devices. Although the current advances in micro-nano fabrication technology have allowed significant integration of many optical elements, such as ultracompact multi-lens objectives [1], high numerical aperture (NA) microlens arrays [2, 3], and other micro-optical elements [4], [5], [6], these devices still rely on classical laws of refraction and reflection and the pixel size of these devices is generally about tens of light wavelengths, leading to limited optical performances.

In recent years, metasurfaces consisting of subwavelength structures have shown great potential to trigger an optical revolution of Engineering Optics from 1.0 to 2.0 [7], which benefits from its unprecedented electromagnetic properties not available in natural materials. It has been shown that the basic properties of light including amplitude, polarization, phase, and frequency can be flexibly controlled in a virtually planar surface by adjusting the geometry shape, material composition, and spatial arrangement of subwavelength structures [8], [9], [10], [11], [12], [13], [14]. Many exotic phenomena and applications have been realized, such as asymmetric photonic spin–orbit interactions [15], [16], [17], [18], [19], [20], [21], [22], [23], [24], spatiotemporal light control [25], [26], [27], [28], multi-dimensional full-color holography [29], [30], [31], [32], [33], [34], [35], [36], [37], [38], invisibility cloaking [39], [40], [41], [42], tunable multifunctional devices [43], [44], [45], [46], [47], [48], [49], [50], miniaturized spectrometers and polarimeters [51], [52], [53], quantum control [54], [55], [56], image differentiation [18, 57], [58], [59], and many others [60], [61], [62], [63], [64].

Besides the aforementioned achievements, metasurfaces have also been successfully applied in the imaging field. Many works and reviews related to metalenses were reported in recent years [65], [66], [67], [68], [69], [70], [71], [72]. Compared to traditional bulk lenses, metalenses are an emerging technology that utilizes subwavelength structures to modulate the phase, amplitude, and polarization of light, and thus exhibit great advantages in the planar configuration, light weight, binary structure, CMOS compatibility, high-NA capability, tunability, polarization selectivity, and so on [73]. For example, one metalens can readily enable a high-NA hyperbolic phase profile to be free from spherical aberration, thus resulting in high diffraction-limited resolution [69]. In contrast, this property is difficult to obtain in traditional optics, especially with a single element, owing to the difficulty in mask an aspherical lens with large curvature [74]. In addition, a lightweight and planar configuration is very suitable for high-resolution telescope imaging and long-distance laser communication requiring a large aperture primary mirror. Objectively speaking, metalenses belong to diffractive elements and still suffer from chromatic aberration [66]. By merging the dispersion propagation phase and dispersion-free geometric phase respectively introduced by the size and orientation difference, broadband achromatic metalenses have been demonstrated in multiple spectral bands ranging from the visible band to the microwave band [75], [76], [77]. Since group delay dispersion achieved by subwavelength structures is limited, up to now the reported ones have a very small aperture or NA [65, 72, 78]. Simultaneous realization of a large aperture and high NA is still a big challenge. Nevertheless, a small-aperture achromatic metalens array has been applied in full-color light-field imaging by Lin et al. [79]. In addition to dispersion control, asymmetric photonic spin–orbit interactions, enabled by the merging of the propagation phase and geometric phase, have also been applied to construct polarization imaging metalenses, showing the ability to identify artificial targets in complex environments [52, 53]. Using the polarization degree of freedom, optical edge detection and three-dimensional imaging metalenses have been demonstrated through various methods [18, 59, 80, 81]. Especially, a single-chip edge-detection metalens without 4F-systems was experimentally demonstrated, suggesting a simpler and more compact edge detection system in practical applications [18].

As a key indicator of imaging systems, the FOV is usually limited by off-axis aberration (especially coma aberration), which makes the cascade of multiple lenses with different materials and shapes necessary for traditional large-FOV imaging systems. In recent years, metalenses have opened a new degree of freedom for addressing the problem of FOV [74, 82], [83], [84], [85], [86], [87], [88], [89], [90], [91], [92], [93], [94]. As a representative example, ultra-large FOV (178°) diffraction-limited imaging has been experimentally demonstrated using a single wide-angle metalens with unprecedented imaging performance regarding the efficiency and angular range [82]. This review concentrates on recent advances of large-FOV imaging technology based on single-chip wide-angle metalenses. The impact of these ultrathin wide-angle metalenses may be substantial in a variety of fields, including large-FOV imaging, beam steering, Fourier optics, and so forth. The review is organized as follows. In Section 2, five basic configurations of sing-chip wide-angle imaging metalenses are briefly introduced. In Section 3, we discuss high-performance wide-angle metalenses from three aspects, including basic requirements and challenges, limitations of traditional methods, and advanced design methods. In Section 4, several representative applications of wide-angle metalenses are reviewed. In Section 5, we give a personal outlook on future potentials and challenges that need to be overcome.

## 2 Mechanisms of wide-angle metalenses

### 2.1 FOV limitation of single-chip metalenses

An ideal planar wide-angle metalens would focus light beams from different incident directions on the same focus plane without aberration. Since the focal position is dependent on the incident angle, the ideal phase of such metalenses should be a function of the incident angle.

**Figure 1:**
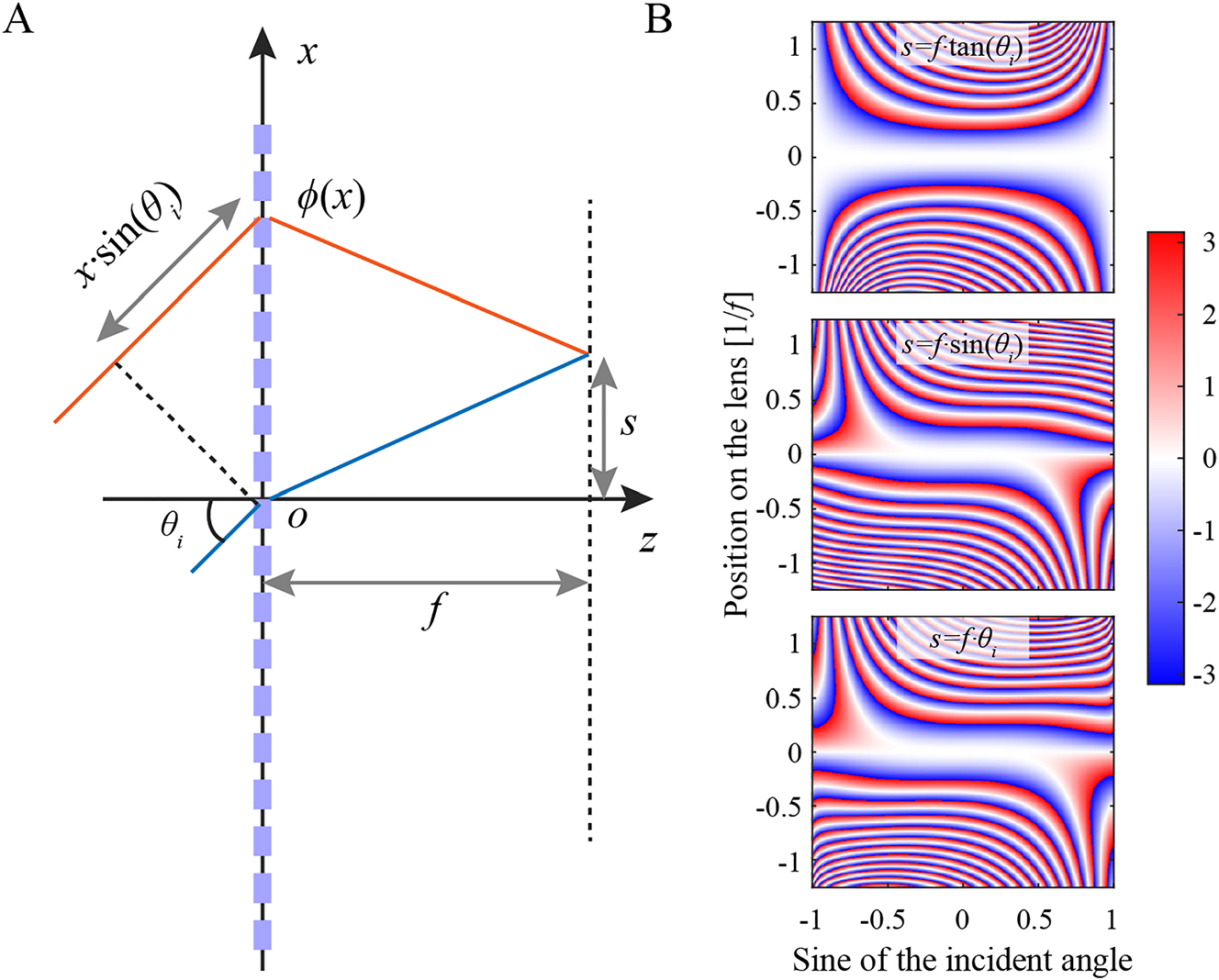
Schematic illustration of perfect wide-angle metalenses and corresponding ideal phase profiles. (A) A flat lens is illuminated with the parallel light incident at angle *θ*_i_ with the optical axis. (B) Ideal phase profiles of perfect wide-angle metalenses. Their corresponding focus offset functions from top to bottom are *s*(*θ*_i_) = *f*·tan*θ*_i_, *s*(*θ*_i_) = *f*·sin*θ*_i_, and *s*(*θ*_i_) = *f*·*θ*_i_, respectively.

For the sake of simplicity, a cylindrical metalens is analyzed and the incident plane is assumed to be the *xz*-plane. As shown in Figure 1A, two light paths with different colors start from an isophase surface of the oblique incident plane wave and end in the focus on the focal plane. To satisfy the condition of the same accumulated phase along the two light paths, the ideal phase profile of the metalens as a function of the incident angle *θ*_i_ at the position *x*, calculated by the method of the equal optical path (well-known Fermat’s principle), can be written as [94]:

(1) $$\phi \left(x,\theta_{i}\right)=-k_{0}\left[x\;\sin\;\theta_{i}+\sqrt{{\left(x-s\left(\theta_{i}\right)\right)}^{2}+f^{2}}-\sqrt{{\left(s\left(\theta_{i}\right)\right)}^{2}+f^{2}}\right]+const.\text{,}$$

where *k*_0_ = 2*π*/*λ* is the wavenumber in free space at the design wavelength of *λ*, *f* is the focal length at the design wavelength, and *s*(*θ*_i_) is the focus offset as a function of the incident angle. Note that Eq. (1) yields a commonly utilized hyperbolical profile when *θ*_i_ = 0, and the ideal phase is dependent on the function of *s*(*θ*_i_) when *θ*_i_ ≠ 0.

Figure 1B shows three typical cases with the focus offset being *f*·tan*θ*_i_, *f*·sin*θ*_i_, and *f*·*θ*_i_, respectively, assuming *f* = 15λ and the aperture equal to 2.5*f*. For the first case, the metalens projects an undistorted image on the image plane. This leads to perfect imaging regardless of resolution, but the corresponding phase profile is strongly dependent on the incident angle. The focus offset properties of the latter two are somewhat similar to Fourier lenses and *f*-theta lenses. Compared with the ideal lens, phase profiles of the latter two are flatter with respect to the incident angle in more regions. Therefore, they are relatively easy to approximate with incident-angle-independent phase profiles [94], although the image distortion exists but can be corrected by later image processing. Nevertheless, the ideal phase is still strongly angle-dependent and asymmetric whatever the focus offset is. In addition, traditional metalens designs usually ignore angular dispersion of phase and even hopes that the phase shift is angle-independent [89, 90, 95], so traditional metalenses have a very limited FOV.

### 2.2 Different configurations for sing-chip wide-angle metalenses

In traditional optics, the FOV of an optical imaging system is expanded by cascading multiple optical elements with the help of the light-ray tracing method, increasing the complexity, volume, and weight of optical systems. The reason for this restriction is that such angle-introduced coma aberration results from broken rotational symmetry of the lens configuration. The well-known pronounced Luneburg lens and compound eyes possess spherical symmetry in the geometry shape, and thus light beams from different directions can also be focused on a spherical surface. Although their FOV can be close or even larger than 180°, the fabrication process of spherical sensors is extremely difficult and the rotational symmetry is not compatible with flat optics as well as current planar fabrication technologies.

**Figure 2:**
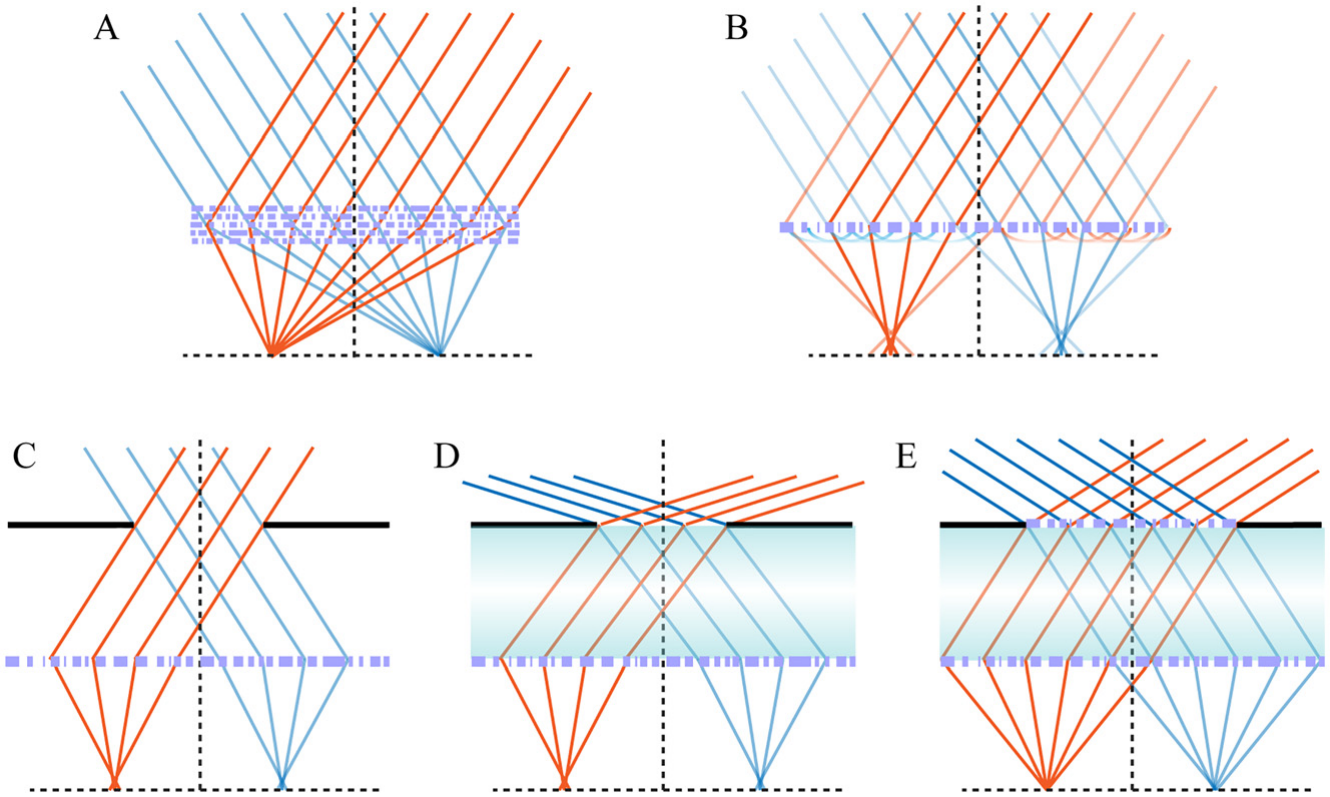
Schematic diagrams of several basic configurations for sing-chip wide-angle metalenses. The red and blue lines represent the light rays with different incidence angles. (A) Optimized multi-parametric geometries. (B) Monolayer quadratic metalens. (C) Landscape quadratic metalens using an air gap. (D) Landscape quadratic metalens using a dielectric gap. (E) Metalens doublets.

In recent years, expanding the FOV of metalenses has attracted increasing attention, because it is one of the critical indicators for imaging applications and can be achieved with the help of the unprecedented electromagnetic control ability of metasurfaces. In special, sing-chip wide-angle metalenses can greatly enhance the integration level of optical systems, showing great potential in surveillance, unmanned vehicles, onboard planes or satellites, medical science, and other new applications [82], [83], [84], [85], [86], [87], [88], [89], [90], [91], [92], [93], [94]. Figure 2 summarizes five basic configurations to correct coma aberration for single-chip metalenses. These methods are mainly divided into two categories, namely the single-structural-side method (top panel of Figure 2) and the double-structural-side method (bottom panel of Figure 2) in this paper, which will be discussed in detail below.

As mentioned above, an ideal phase profile free from off-axis aberration is necessarily angle-dependent. Therefore, the most direct way to build perfect wide-angle metalenses is to control the angular dispersion of meta-atoms. To improve degrees of freedom for angular phase control, multi-parametric geometries and advanced design methods are necessary. For example, Lin et al. proposed a general topology-optimization framework for inverse design of multilayered wide-angle metalenses [90], as illustrated in Figure 2A. As a concept-proof, a near-perfect wide-angle metalens composed of five layers of topology-optimized aperiodic silicon gratings was numerically demonstrated with an effective NA of 0.35, a focal length of 30*λ*, and a FOV of 40°. The simulated phase profile showed good agreement with the ideal one at several optimized half FOVs (0°, ±7.5°, ±15°, and ±20°). In addition, an epsilon-greedy algorithm-based scheme was proposed by Hao et al. to optimize aberration-compensated flat lenses, which is composed of two layers of nanoring structures contributing to focusing and off-axis aberration compensation, respectively [86]. A wide-angle multistep flat lens was demonstrated with an effective NA of 0.45, a focal length of 1 mm, and a FOV of 32° × 32° at the wavelength of 633 nm, but both point spread function and modulation transfer function are still somewhat far from the corresponding diffraction limit. Therefore, how to optimize large-area wide-angle metalenses with continuous diffraction-limited FOVs is still an open question, because both of them would greatly increase the burden of time-consuming bruteforce numerical solvers.

**Figure 3:**
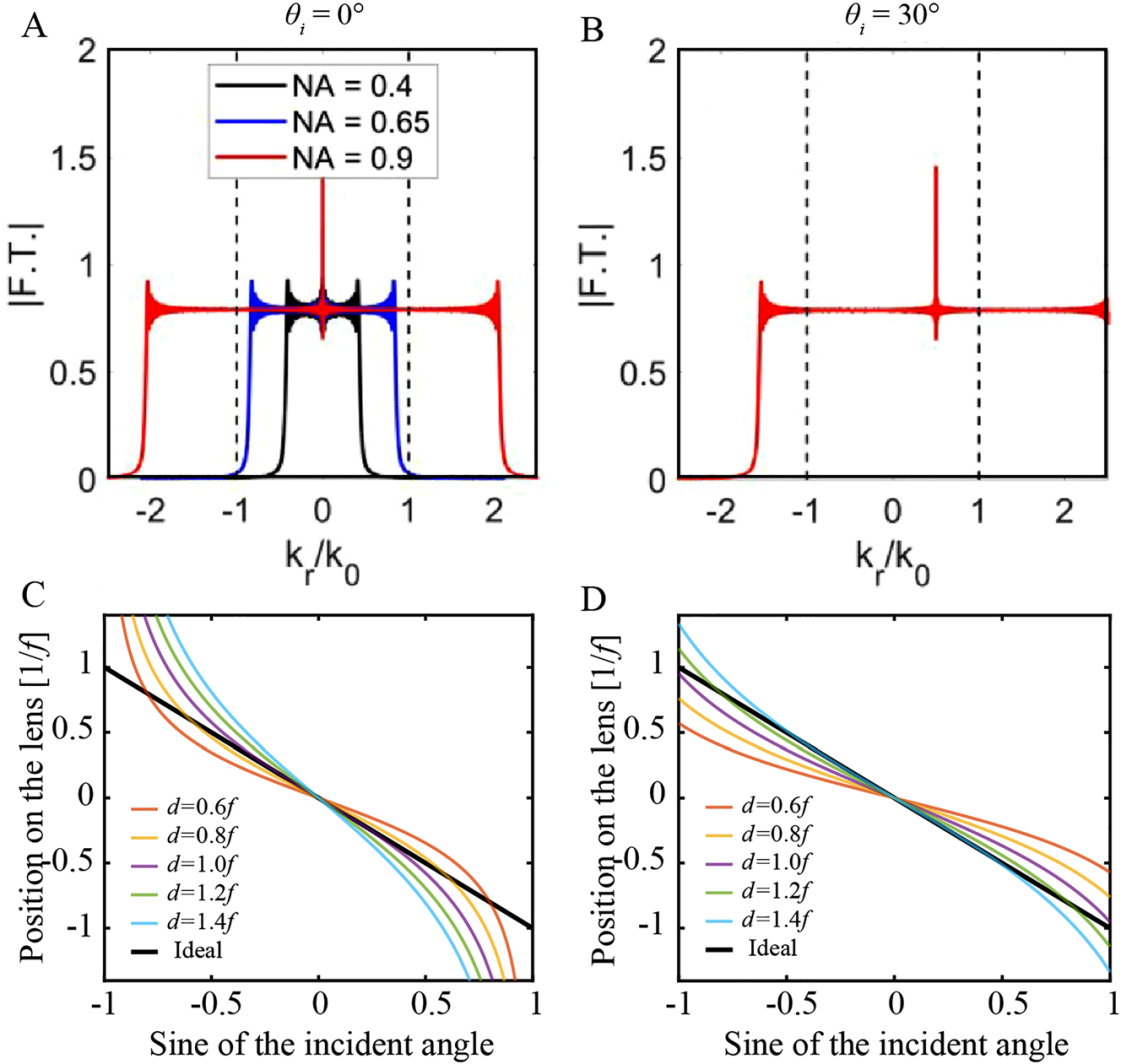
Performance of quadratic metalenses. (A) Fourier transform spectra of the wide-angle metalens for an NA of 0.4 (black), 0.65 (blue), and 0.9 (red), for normal incidence. (B) Same as the red line in (A), but for oblique incidence. Adapted with permission from ref. [85]. Copyright 2020, American Chemical Society. (C–D) Position of the light axis projected onto two kinds of landscape quadratic metalenses after passing through the aperture stop for the configuration of Figure 2C and E, assuming that the refractive index of the dielectric gap is equal to 1.45.

Another single-structural-side method is to use symmetry transformation of quadratic metalenses from rotational symmetry to translational symmetry. This new concept of symmetry transformation was proposed by Pu et al. for the first time in 2017 [74]. Since the phase profile of such wide-angle metalenses follows a quadratic form, it was also termed as “quadratic metalens”. Assuming that the incident collimated light beam lies in the *xz*-plane with an arbitrary incident angle of *θ*_i_ to the normal axis of the quadratic metalens, the phase carried by outgoing light should be:

(2) $$\phi \left(r,\theta_{i}\right)=-\frac{k_{0}}{2f}r^{2}-k_{0}x\;\sin\;\theta_{i}=-\frac{k_{0}}{2f}\left[{\left(x+f\;\sin\;\theta_{i}\right)}^{2}+y^{2}\right]+\frac{k_{0}f\sin^{2}\theta_{i}}{2}\text{,}$$

where *k*_0_ = 2*π*/*λ* is the wavenumber in free space at the design wavelength of *λ*; *f* is the focal length at the design wavelength; *kx*sin*θ*_i_ is the gradient phase introduced by the oblique incidence. Since the last term in the right hand is independent of the coordinate and can be ignored, each incident angle of *θ*_i_ corresponds to a focus in the corresponding focal plane, with a focus offset of −*f*·sin*θ*_i_. Thus, the rotational effect of the oblique incidence light is perfectly converted to the translational symmetry of the focus, as shown in Figure 2B. The physical properties of such wide-angle metalenses were further discussed through Fourier analysis by Martins et al. [85]. The Fourier Transform spectrum of the quadratic metalens is flat enough, which guarantees its symmetry at oblique incidence ignoring evanescent components. As shown in Figure 3A and B, for an NA ≈ 0.9 the Fourier Transform covers the range of normalized *k*_*0*_ vectors from −2 to 2, which makes the Fourier Transform invariant for oblique incidence, resulting in a FOV of ∼180°. Here, NA is defined as *D*/(*D* ^2^ + 4*f* ^2^)^½^, where *D* is the diameter of the metalens. Note that it differs from the effective NA. Furthermore, an in-depth analysis of imaging properties of quadratic metalenses was also provided by Lassalle et al. [83]. This work underlined the importance of considering barrel distortion in combination with the optical resolution limit and/or cut-off frequency of detectors when designing quadratic metalenses for imaging configuration, especially for a short focal length.

When the quadratic metalens directly faces the object, the effective aperture can reach the maximum but at the cost of large spherical aberration, resulting in strong background noise and low imaging contrast. Therefore, the second category of FOV expanding methods requires an additional aperture stop in the front of metalenses, so we refer it to the double-structural-side method. It is an ancient and common method to compensate for coma aberration in traditional optics. For example, the classical landscape lens is based on this principle. An early study showed that a simple system consisting of a single planar diffractive lens with an aperture stop can correct the coma, astigmatism, and field curvature and thus provides a (monochromatic) imaging performance superior to conventional systems consisting of several lens elements [96]. Recent studies showed that ultra-large FOV with considerable imaging resolution can be realized by the quadratic metalens incorporated with an aperture stop [82, 84, 87, 88, 92, 93].

Specifically, this method can be subdivided into three types, as illustrated in Figure 2C–E. An easy way to compress spherical aberration within the tolerance of the metalens is to place an aperture stop in front of the metalens with an air gap, as shown in Figure 2C. For example, a near-infrared large-FOV metalens was proposed by Engelberg et al. via putting a 1.35 mm aperture stop in front of a quadratic metalens with a focal length of 3.36 mm [88]. With the help of the aperture stop, a near-diffraction-limited FOV of 40° × 40° was realized at the wavelength of 800 nm in the design. However, simply placing an aperture stop in front of the quadratic metalens can only support a limited diffraction-limited FOV, because the actual position (−*d*·tan*θ*_i_) of the light axis projected onto the quadratic metalens after passing through the small hole cannot match well with the ideal position (−*f*·sin*θ*_i_), where *d* indicates the distance between the aperture stop and the quadratic metalens. This issue can be well addressed via putting the metalens and aperture stop on two sides of a high-index transparent plate, as shown in Figure 2D. In this case, after optimizing the thickness *d*, the actual of $-d\;\sin\;\theta /\sqrt{n^{2}-\sin^{2}\theta_{i}}$ mathematically gets closer to −*f*·sin*θ*_i_ in a wide angular range, when the refractive index *n* is larger than 1. For example, a diffraction-limited metalens with a record FOV of 178° × 178° at the wavelength of 940 nm has been demonstrated by two groups using a 5 mm thick silica plate [82] and a 3.9 mm thick sapphire plate [84], respectively. The smaller the aperture stop, the closer the quadratic phase is close to the hyperbolic phase. As a result, the effective NA of wide-angle metalenses based on Figure 2D is around 0.2 [82, 84, 87], because only a small area can approximate with an incident-angle-independent phase profile, as illustrated in the middle panel of Figure 1B. Strictly speaking, it just means that the aberration is within the tolerance of the metalens for the two configurations shown in Figure 2C and D. One can employ metalens doublets to correct spherical aberration and thus increase the effective NA at the cost of higher fabrication complexity [92, 93], as shown in Figure 2E. Using this method, a miniature metasurface doublet camera has been proposed with an effective NA of 0.49 and a FOV of 60° × 60° [16]. However, the effective NA needs to be compromised if a larger FOV is required. This trade-off may be alleviated via properly using angular dispersion properties of special subwavelength structures.

## 3 Challenges of high-performance wide-angle metalenses

### 3.1 Basic requirements and challenges

**Figure 4:**
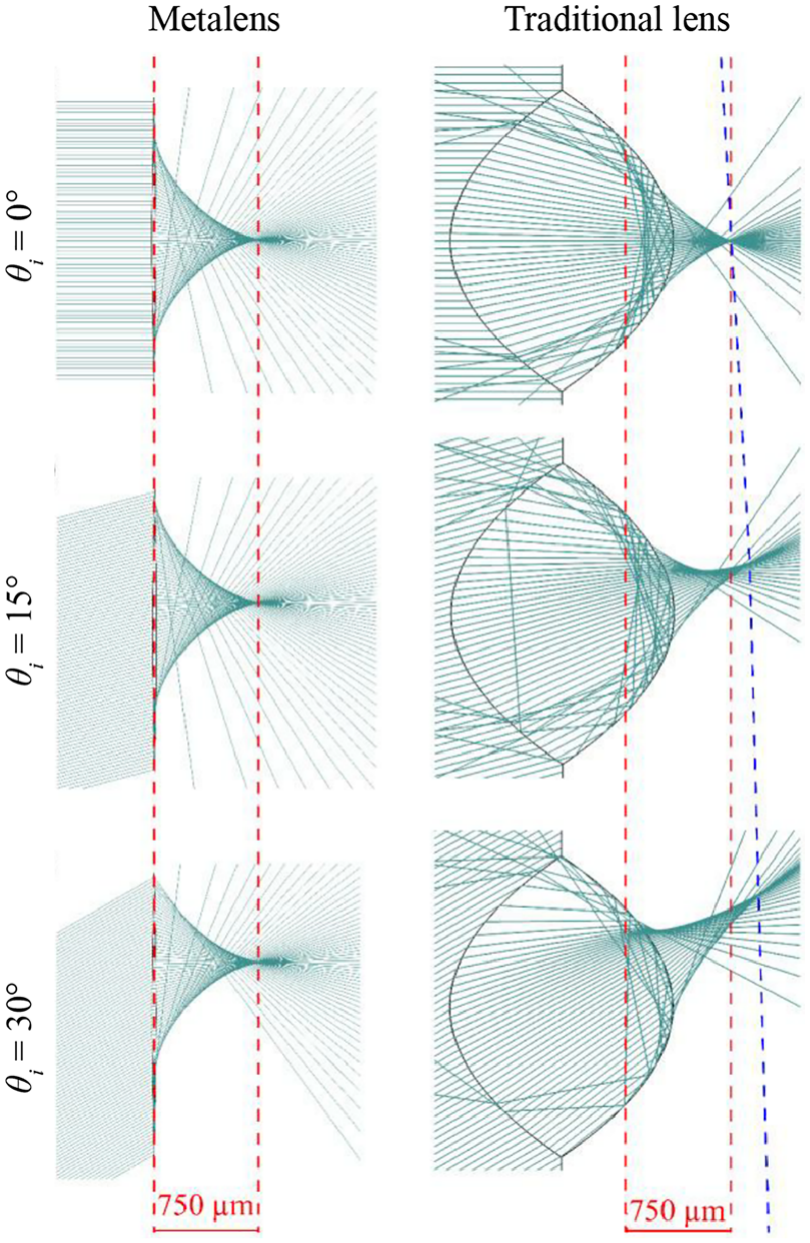
Comparison between the wide-angle metalens (first column) and an equivalent bulk quadratic lens (second column) at different angles. The red dashed lines mark lenses’ output aperture and focal position at normal incidence, which coincides with oblique incidence for the wide-angle metalens. The blue dashes line marks the focal position of the bulk lens for different angles of incidence. The two lenses have the same focal length of 750 μm with NA = 0.8. Adapted with permission from ref. [85]. Copyright 2020, American Chemical Society.

Although the principle of wide-angle metalenses seems straightforward, the aforementioned symmetry transformation could not be easily realized with a traditional refractive or diffractive lens. As demonstrated by Martins et al., the wide-angle metalens can focus light beams from different directions at the same distance, but the focal length of the bulk quadratic lens, in contrast, is dependent on the angle of incidence, as shown in Figure 4. This effect is schematically highlighted by comparing the blue dashed line with the red dashed line in the right panel of Figure 4. These two lines mark the focal point for different angles of incidence and the focal distance at normal incidence, respectively. To further prove the point that the optical performance could not be realized through a bulk lens, one can readily deduce [85]:

(3) $$\phi_{\text{sph}}\left(r\right)=\lim_{\begin{array}{l} \left(R,\Delta n\right)\to \left(+\infty ,+\infty \right)\\ \begin{array}{ll} f & const. \end{array} \end{array}}-2k_{0}n_{ext}\Delta n\left(R-\sqrt{R^{2}+r^{2}}\right)=-\frac{k_{0}r^{2}}{2f}n_{ext}\text{,}$$

where *n*_ext_ is the refractive index of the focusing media; Δ*n* is the difference in refractive indices between spherical lens media and focusing media; *R* is the spherical lens radius of curvature. As can be seen from Eq. (3), one metalens with a quadratic phase profile corresponds to the limit of a spherical lens with infinite curvature radius and infinite refractive index. As a result, it is concluded that, if near 180° FOV is required, the wide-angle metalens has no bulk counterpart and a flat design is necessary [85].

Next, we will explain why the wide-angle metalens can hardly be realized by traditional diffraction elements, which implement a 2*π*-phase delay across the component surface by a *ϕ*(*r*)*λ*/[2*π*(*n* − 1)] thickness profile, such as the well-known “échelette” blazed grating. Nowadays, échelette-type diffractive elements can be manufactured at low cost by replication technologies including embossing, molding, and casting [68]. However, the discontinuous of wrapped phase introduces a shadow, leading to light waste into undesired diffraction orders, especially for short period échelettes. For the quadratic phase profile, the effective transverse wavevector induced by the phase gradient is quite large, i.e., *∂* *ϕ*/*∂* *r* = 1.2*k*_0_ at *r* = 1.2*f* for normal incidence. Such a big phase gradient makes the period smaller than the operating wavelength, and thus strong diffraction effect would bring obvious phase error. Even using metasurfaces composed subwavelength substructures, it is still difficult to build high-performance wide-angle metalenses, which will be discussed in the next section.

### 3.2 Limitations of traditional methods

**Figure 5:**
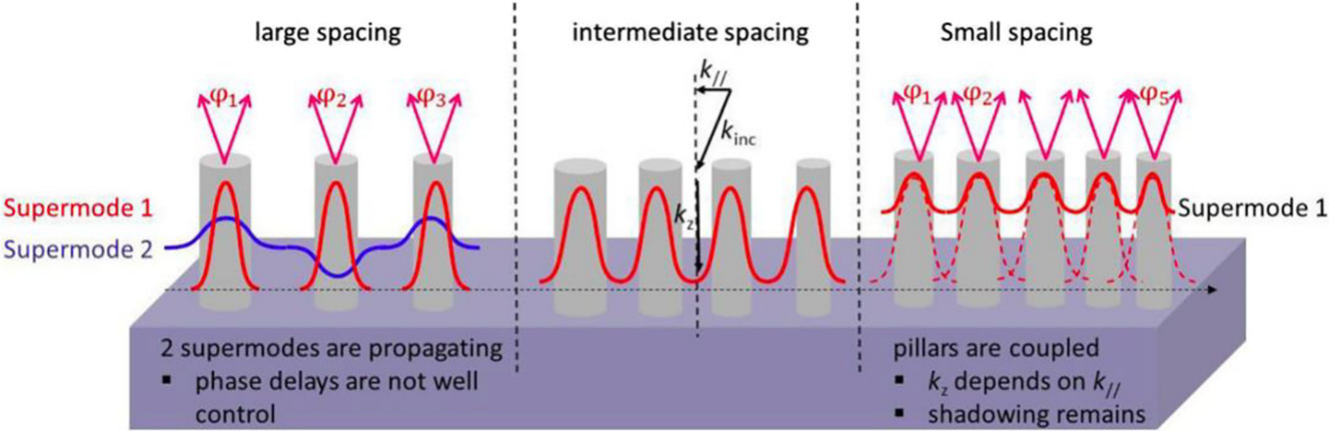
Critical choice of the unit spacing in waveguide-type structures. Left panel: large spacing. The phase delay that depends on both modes is hard to be accurately controlled. Central panel: intermediate spacing. The nanostructures are uncoupled and the phase delay is independent of k_//_. Right panel: small spacing. The phase sampling is fine but the nanostructures are coupled electromagnetically. Adapted with permission from ref. [68]. Copyright 2017, WILEY-VCH.

In general, metalenses are composed of subwavelength structures that are usually arranged in the tetragonal lattice or the hexagonal lattice. Both of them face the same problem of insufficient phase sampling over limited space, especially for large-NA metalenses. This issue lies in the trade-off between phase sampling and electromagnetic coupling. According to the diffraction theory, the diffraction efficiency depends on the discrete level *N* of quantized phase distributions and can be given as [(*N*/*π*)sin(*π*/*N*)]^2^ [97]. This equation indicates that for 2, 4, 8, and 16 phase quantization levels, the diffraction efficiency will be 40.5%, 81.1%, 95.0%, and 98.7%, respectively. Increasing the phase sampling by the smaller spacing of unit cells seems to be an easy solution to improve diffraction efficiencies. However, the small unit spacing will enhance electromagnetic coupling among adjacent structures, even though for waveguide-type nanostructures with strong electromagnetic confinement. Figure 5 given by Lalanne et al. summarizes the main design constraints on the spacing values for both propagation-phase and geometric-phase waveguide-type metalenses [68]. When the phase sampling is fine (right panel), the supermode propagation constant *k*_*z*_ changes with the parallel wavevector *k*_//_ of incident light owing to the strong electromagnetic coupling, and there is still the shadow effect. In contrast, when the spacing is too large (left panel), two propagating supermodes make the phase delay hard to accurately controlled, especially when the incident angle varies. Accurate control of the phase delay requires monomode propagation for light in the nanostructures whose spacing should be smaller than the structural cutoff, where the structural cutoff is defined as the period above which the periodic structure no longer behaves like a homogenous thin film and below which only one propagating mode is supported [98]. In the central panel, the spacing is appropriate and the normalized propagation constant is equal to the effective index of the isolated waveguide. Thus, these nanostructures are uncoupled and the phase delay becomes independent of the incident angle, resulting in strong angular robustness. It is showed that the intermediate spacing usually chooses spacing-to-wavelength ratios ranging from 0.4 to 0.5 [98, 99]. In this case, the big phase gradient of the wide-angle metalens causes the fact that the discrete level of the wavelength at the edge is less than 2, resulting in extremely low diffraction efficiencies and strong scattering noise.

As discussed above, electromagnetic coupling among adjacent subwavelength structures is the main factor for the difficulty in achieving large phase gradients with high efficiency. Someone may think that this issue can be avoided as long as the influence of electromagnetic coupling is considered in the simulation, but it is unsubstantial for the traditional design method of metalenses or more general metasurface devices. In conventional metasurface designs, subwavelength structures optimized by parameter scanning and manual selection are stitched together to produce a desired phase profile response, where Bloch or periodic boundary conditions are assumed. If the phase gradient is small, the size gradient is also so small that the periodic assumption almost holds. However, for high-NA metalens, significant local aperiodicity would cause unpredictable differences in electromagnetic response (especially for phase shifts) between the periodic unit cell and the aperiodic full model [100]. As a result, it is still hard to design high-performance wide-angle metalenses with traditional methods. In the next two subsections, two advanced methods, namely adjoint optimization and catenary optics, will be discussed, which can or have great potential to overcome the aforementioned limitations.

### 3.3 Advanced design methods for wide-angle metalenses

#### 3.3.1 Adjoint optimization

Traditional metasurface design belongs to a forward problem, in which, a given geometry and set of conditions correspond to a unique electromagnetic response. To overcome the limitations of traditional methods mentioned above, it is common to attempt a slightly modified inverse problem: which geometry is closest to achieving the desired electromagnetic response [101]. In this case, the inverse problem becomes an optimization problem. Many optimization algorithms like the well-known genetic algorithm and particle swarm optimization algorithm have been proposed to modulate the local size of metasurface devices [102], [103], [104], [105], [106]. However, these optimization algorithms usually need hundreds of populations in a single iteration, which causes the problems of the huge amount of calculation and low design efficiency. Adjoint optimization is a versatile and powerful technique that has been extensively exploited to design high-performance metasurface devices [107], [108], [109], [110]. Adjoint optimization calculates Maxwell’s equations twice in every iteration operation [111], offering a potential inverse design approach with high design efficiency. The most important advantage of adjoint optimization is that only two simulations, namely forward and adjoint simulations, are required to get the gradient of all variables relative to the single-objective figure of merit, and thus its design efficiency is independent of optimization variables [101]. For multi-objective optimization, each objective function usually requires two simulations to understand how structural changes can increase the total figure of merit. According to the way of structural deformation, adjoint optimization can split nicely into two categories: topological optimization (topological deformation, i.e., new inclusions) and shape optimization (shape deformation, i.e., boundary movements) [101]. Here, we briefly review some high-performance metasurfaces that are designed using adjoint optimization.

**Figure 6:**
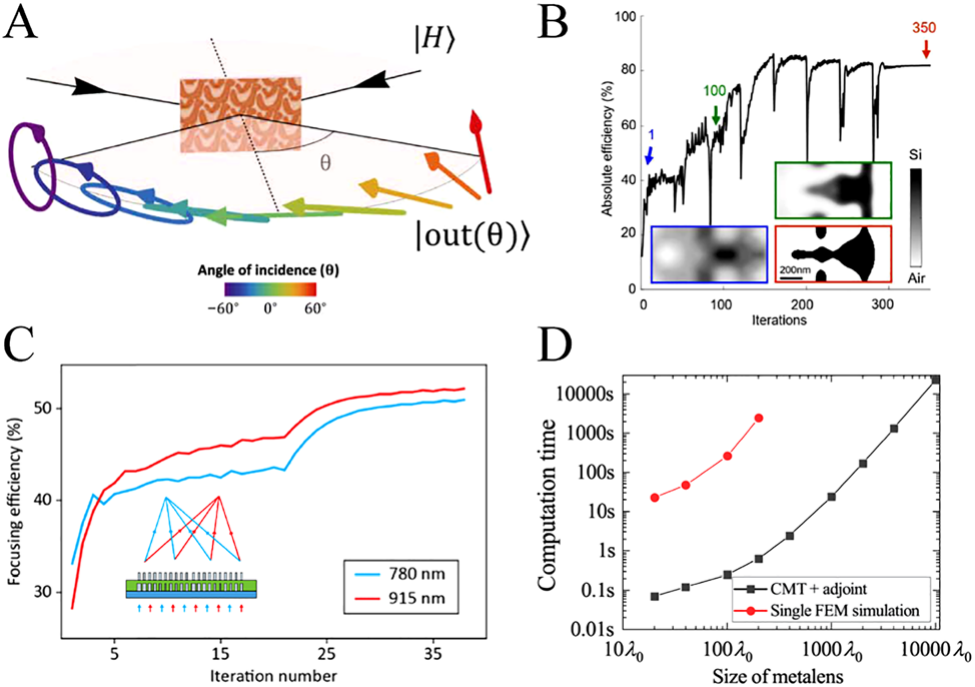
Some metasurface devices based on adjoint-based topological and shape optimization. Schematic of continuous angle-tunable birefringence. Adapted with permission from ref. [112]. Copyright 2020, American Association for the Advancement of Science. (B) Plot of deflection efficiency during the topological optimization process for the freeform metagrating with a deflection angle of 75°. Adapted with permission from ref. [113]. Copyright 2017, American Chemical Society. (C) Evolution of the focusing efficiencies over the course of the shape optimization. Adapted with permission from ref. [100]. Copyright 2020, Optical Society of America. (D) Computation time of single finite element method simulation and one iteration using an approach combined with coupled-mode theory (CMT) and shape optimization. Adapted with permission from ref. [114]. Copyright 2021, American Chemical Society.

Adjoint-based topological optimization usually aims to optimize the permittivity distribution and has been extensively exploited to design freeform metasurfaces with non-intuitively quasi-continuous topological shapes. For example, Piggott et al. experimentally demonstrated a freeform on-chip wavelength demultiplexer that can split 1300 nm and 1550 nm light from an input waveguide into two output waveguides, showing low insertion loss, low crosstalk, and wide bandwidth [115]. Z. Shi et al. proposed the concept of continuous angle-tunable birefringence with freeform metasurfaces [112]. As shown in Figure 6A, it can be continuously adjusted from linear to elliptical birefringence by changing the angle of incidence. Since the freeform structures have no limitation of phase sampling, adjoint-based topological optimization has shown great potential to design large-angle deflectors and high-NA metalenses [113, 116], [117], [118]. As illustrated in Figure 6B and D. Sell et al. demonstrated large-angle freeform metagratings that can deflect light to angles as large as 75° with efficiencies higher than 80%, showing great advantages compared with traditional discrete structures [113]. After that, Kim et al. demonstrated wide-angle two-dimensional diffractive metagratings whose power distribution of diffraction orders can be arbitrarily tailored, showing great application potential in three-dimensional imaging [117]. In addition to periodic metagratings, adjoint-based topological optimization has also been used to design high-NA broadband achromatic metalenses, with an average efficiency being around 40% over 450–700 nm wavelength range for NA = 0.9 [118]. Adjoint-based topological optimization has high degrees of freedom but at the cost of high difficulty in parameterizing structural shape and more iteration for binarization.

In contrast, adjoint-based shape optimization does not change the basic shape, and the deformation of the structure boundary is changed along its normal direction every iteration operation, resulting in easier quantized compensation of fabrication error [119]. Another important advantage is that the initial structure of adjoint-based shape optimization can be a binary structure. In this case, an optimized binary structure obtained by manual or computer optimization can be selected as the initial one. Therefore, the optimization parameters can be flexibly adjusted to further improve the performance, because the performance of the final structure could be guaranteed not worse than that of the initial one. In contrast, the initial structure of topological optimization requires a continuous permittivity distribution, and thus an artificially optimized binary structure cannot be directly utilized as the initial one [120]. As a result, adjoint-based shape optimization has recently attracted increasing attention in metalens design [100, 119, 121, 122]. For example, compared with monolayer metasurfaces, multilayer metasurfaces provide more degrees of freedom and can be utilized for the implementation of multifunctional devices, but the traditional design method usually assumes layers to be non-interacting and ignores inter-layer interactions [123, 124], losing part of degrees of freedom. In contrast, Mansouree et al. experimentally demonstrated 2.5D dual-wavelength achromatic metalenses, in which both inter-post and inter-layer electromagnetic couplings were accurately accounted for, with average focusing efficiency increased from ∼30% to ∼50% through adjoint-based shape optimization [100], as shown in Figure 6C. More recently, Liu et al. proposed a fast design of high-NA polarization-multiplexing metalenses with a uniform array as the initial structure, and its diffraction efficiency can reach about 92% after about 20 iterations [122].

The adjoint optimization technique provides an effective approach for overcoming the design limitations of wide-angle metalenses, but it is also limited by time-consuming bruteforce numerical solvers to simple demonstrations of either very small or period devices. Current computer technology cannot cope with large-area electromagnetic simulations. To address this limitation, Phan et al. proposed a strategy for optimizing large-area metalenses by stitching together individually optimized sections [125]. A curvilinear phase profile was divided into a series of linear sections that were about 3*λ* wide and optimized in isolation. This method enables the optimization of large-area devices; however, linear approximation and individual optimization do not accurately model the local response of high-NA metalenses, resulting from the fact that near-field coupling between sections cannot be considered fully. Recently, adjoint optimization has been combined with other fast-approximate solvers for inverse design of high-NA and large-area metalenses [114, 121]. As shown in Figure 6D, the combination of CMT and adjoint optimization can enable the inverse design of a metalens with a size up to 10^4^ *λ*, but the largest size of approximately 200*λ* is allowed for finite element method simulations [114]. However, these methods still require direct or indirect full-model simulations many times and thus are still very time-consuming. Another important issue required to be further addressed is the initial structure. Mathematically, adjoint optimization is based on the method of gradient descent, in which the potential to yield exceptional devices after a large number of iterations depends to some extent on the initial structure [101]. However, there currently are no effective and predictable methods for specifying good initial structures for adjoint optimization [120], although high-performance devices were demonstrated in prior work using random initial structures or manually optimized initial structures.

#### 3.3.2 Catenary optics

Using catenary-like quasi-continuous structures is another effective method for designing high-performance wide-angle metalenses. Catenary structures were first introduced from architecture to subwavelength optics by Pu et al. in 2015 [126]. After that, a new research direction of catenary optics was developed and has been extensively exploited [127], [128], [129], [130], [131]. Owing to the ability of quasi-continuous wavefront control, catenary structures have attracted increasing attention for a large number of applications, such as high-efficiency accelerating beam generation [132, 133], tunable circular-polarization beam splitting [134], polarization-controlled unidirectional excitation of surface plasmon polaritons [135], wide-angle imaging [82], and many others. One can see a recent review for the history, basic theories, functional devices, and applications of catenary optics and catenary electromagnetics [127]. In this section, we briefly introduce how to construct a high-performance catenary-based wide-angle metalens without the requirement of extensive numerical simulations.

**Figure 7:**
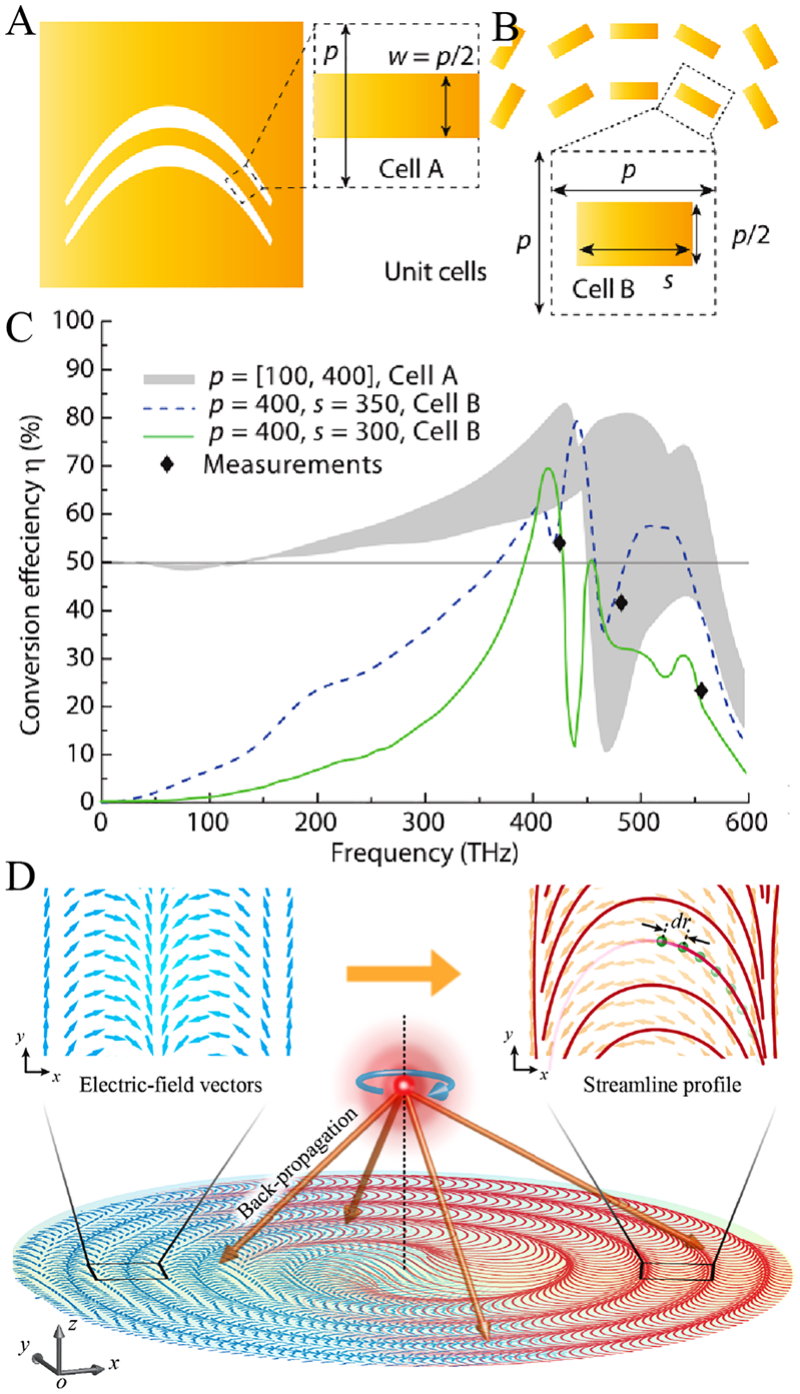
Comparison of the conversion efficiency between the catenary structure and discrete nanostructure. (A) and (B) Schematic diagrams of unit cells in the catenary structure (A: *s* = *p*) and discrete nanostructure (B: *s* < *p*). (C) Simulated conversion efficiency of the unit cell A and unit cell B. (D) Concept illustration of catenary-like streamline metalenses. (A–C) Adapted with permission from ref. [126]. Copyright 2015, American Association for the Advancement of Science. (D) Adapted with permission from ref. [82]. Copyright 2021, WILEY-VCH.

First, we start from catenary structures with equal phase gradients to explain the basic principle and advantages of catenary structures. Catenary functions have two basic forms, i.e., the ordinary catenary and the catenary of equal strength, which were formulated in 1691 and 1826, respectively [127]. The ordinary catenary has the form of hyperbolic cosine, and the catenary of equal strength can be given as:

(4) $$y=\frac{\Lambda }{\pi }\ln\left(\left|\sec\left(\pi x/\Lambda \right)\right|\right)\text{,}$$

where Λ is the horizontal length of the catenary. Under the illumination of circularly polarized (CP) light, the spin-flipped component of output light fields will carry well-known Pancharatnam–Berry phase (also called geometric phase) [136], which can be given as −2*σξ*(*x*, *y*), where *σ* = ±1 denotes left- and right-handed CP light and *ξ*(*x*, *y*) indicates the rotation angle of an anisotropic structure. Because the inclination angle of such curves has a form of *ξ*(*x*, *y*) = tan^−1^(d*y*/d*x*) = *πx*/Λ, there is a linear and dispersion-free phase distribution of *ϕ*(*x*) = −2*σπx*/Λ for catenary structures. It should be noted that the two ends of the catenary should be truncated because the value of Eq. (4) is infinite for *x* = ±Λ/2. One merit of catenary structures over traditional discrete metasurfaces is the quasi-continuous phase distribution; thus, a much larger phase gradient and better performance are possible. As shown in Figure 7C, the unit cell of the catenary structure can allow higher conversion efficiency and bandwidth than that of traditional discrete metasurfaces. It is worth noting that the two unit cells shown in Figure 7A and B only differ from each other in the length of the metallic bar. Recently, during the optimizing large-angle metagratings by adjoint-based topological optimization, Xu et al. successfully observed the topological transformation process from discrete structures to catenary-like quasi-continuous structures [116], which can further prove that the quasi-continuous metasurface outperforms the discrete one.

Inherent advantages in realizing a large phase gradient make catenary structures very suitable for constructing wide-angle metalenses. A general method for constructing catenary-like streamline metasurfaces with any continuous phase distribution was proposed recently [82]. As shown in Figure 7D, one metalens that focuses left-handed CP incidence into a tight spot is taken as an example. One can obtain the electric field (blue arrows) on the *xy*-plane (*z* = 0) by time reverse, which is given as *E*_*x*_ = exp[i*ϕ*(*x,y*)] and *E*_*y*_ = iexp[i*ϕ*(*x,y*)], where *ϕ*(*x,y*) indicates the time-reversed phase distribution. According to the principle of geometric phase, such a field could be generated by a spatially variant half waveplate under the illumination of the left-handed CP light. Mathematically, the continuous main axes (orange arrows) of the half waveplate could be seen as the streamlines of a new vector field defined by *U*_*x*_ = exp[i*ϕ*(*x,y*)/2] and *U*_*y*_ = iexp[i*ϕ*(*x,y*)/2]. Then, the local slope of such an auxiliary vectorial field is determined by a differential equation:

(5) dydx=Re(Uy)Re(Ux)=−tan[ϕ(x,y)/2].

Subsequently, numerical integration is employed to obtain the coordinate of the catenary-like streamline (red curves):

(6) $$\begin{array}{l} x_{m+1}=x_{m}+\cos\left[\phi \left(x_{m},y_{m}\right)/2\right]\text{d}r\\ y_{m+1}=y_{m}-\sin\left[\phi \left(x_{m},y_{m}\right)/2\right]\text{d}r\text{,} \end{array}$$

where d*r* indicates the integral step length and should be small enough. By appropriately choosing the starting/end points, the whole plane can be filled with catenary-like streamlines with desired density.

After that, the catenary-like streamlines should be converted into solid shapes. For metallic materials, one can readily construct a solid structure or an aperture by two same streamlines with a proper shift, but they suffer from the ohmic loss. For loss-free dielectric materials, there would be a parasitic propagation phase gradient that is not required, owing to the spatially variant local equivalent period of these catenary-like streamlines. It is worth noting that there is almost no propagation phase gradient for metallic streamline structures exist, because of their ultra-thin thickness. To avoid the existence of the parasitic phase gradient, which would enhance scattering noise, an isophase streamline optimization strategy was proposed to realize pure geometric phase modulation [82]. The key of this method is to spatially vary the width of dielectric streamline structures by resorting to a database of the local period, width, and propagation phase. Such a database can be built by simple numerical simulations, so the design time is almost independent of the area. As a result, a centimeter-scale wide-angle metalens was experimentally demonstrated with the maximum diffraction efficiency approached 100% in ultra-wide spectral and angular ranges.

## 4 Representative functional devices and applications

### 4.1 Flat imaging with an ultra-large FOV

**Figure 8:**
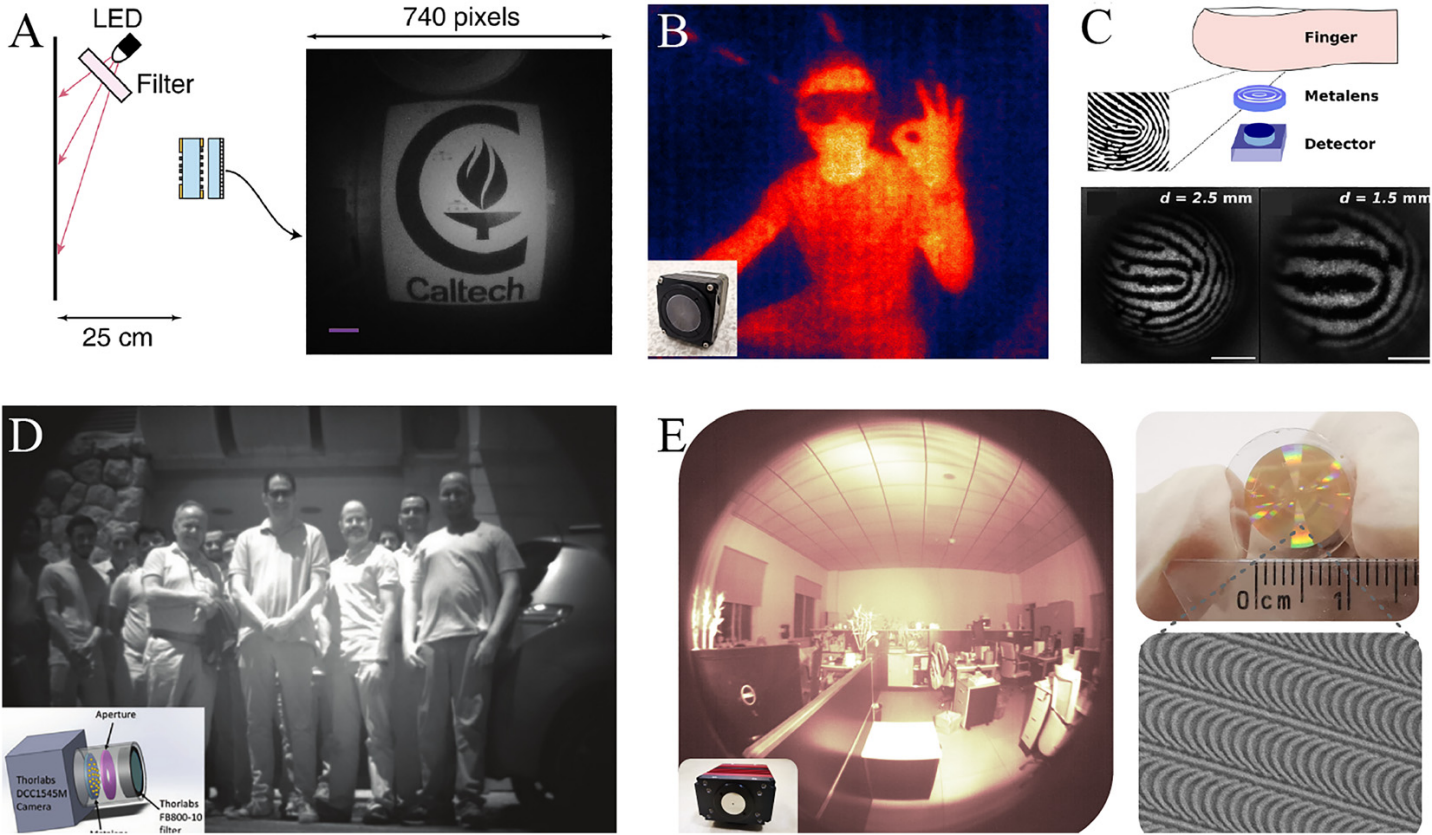
Applications of wide-angle metalenses for flat large-FOV imaging. (A) Imaging setup and the image captured by the miniature metasurface doublet camera. Scale bar: 100 μm. A bandpass filter (850 ± 5 nm) was placed in the front of the LED to reduce the chromatic aberration. Adapted with permission from ref. [93]. Copyright 2016, Distributed under the Creative Commons Attribution 4.0 International License (CC BY). (B) A human thermal image formed by the long-wave infrared metacamera. Adapted with permission from ref. [82]. Copyright 2021, WILEY-VCH. (C) Artistic picture of the fingerprint capturing device applied for fingerprint detection. Scale bar: 100 μm. Adapted with permission from ref. [83]. Copyright 2021, American Chemical Society. (D) An outdoor image formed by the near-infrared metacamera with a FOV of 40° × 40°. Adapted with permission from ref. [88]. Copyright 2020, Distributed under the Creative Commons Attribution 4.0 Public License (CC BY). (E) An indoor image formed by a near-infrared metacamera with a FOV of 178° × 178°. The picture of the metacamera is shown at the bottom left with optical and SEM images of the wide-angle metalens shown in the right panel. Adapted with permission from ref. [82]. Copyright 2021, WILEY-VCH.

One representative application of wide-angle metalenses is flat imaging with an ultra-large FOV. Owing to the advantages of ultra-thin and planarization, the wide-angle metalens can be integrated with a sensor for realizing a planar configuration. Figure 8A shows the imaging setup and corresponding image captured by a miniature metasurface doublet camera proposed by Arbabi et al. in 2016 [93]. This camera has an effective NA of 0.49, a pupil diameter of 0.8 mm, a FOV of 60° × 60°, and operates at 850 nm wavelength with 70% focusing efficiency. The total dimensions of the camera are 1.6 mm × 1.6 mm × 1.7 mm, showing a high level of integration. After that, meta-lens doublet in the visible, which has an effective NA of 0.44, a pupil diameter of 0.31 mm, and a FOV of 50° × 50°, was demonstrated by Groever et al. in 2017 [92]. It could operate at wavelengths from 470 nm to 660 nm with focusing efficiencies ranging from 25% to 50%. It is noted that the focal length of this meta-lens doublet changes with the operating wavelength owing to the existence of chromatic aberration. As a result, a narrowband light source is still necessary.

Compared with meta-lens doublets, monolayer quadratic metalenses are easier to be implemented and do not need to consider the alignment of two layers. In 2021, a highly integrated long-wave infrared metacamera, composed of a quadratic metalens (focal length: 6.5 mm; diameter: 25.4 mm), an infrared band-pass (10.6 μm ± 125 nm), and an infrared detector, was reported [82]. Owing to the high performance of streamline structures obtained by the isophase streamline optimization, this metacamera had an angular-independent diffraction efficiency of around 90% and was directly used for human thermal imaging with the FOV larger than 140° × 140°, even if a narrow-band filter was embedded and the infrared detector was uncooled. As shown in Figure 8B, the body contour and gesture were clearly captured, and the resolution could reach the cut-off spatial frequency of the employed infrared detector. In the same year, the application of fingerprint detection was experimentally demonstrated by Lassalle et al. using the quadratic metalens [83]. The quadratic metalens has a diameter of 0.5 mm, a focal length of 203 μm, and an operating wavelength of 740 nm. As shown in Figure 8C, a whole 5 mm × 5 mm fingerprint with features of ∼100 μm can be captured by a quadratic metalens at a distance of 2.5 mm under the illumination of laser light. In this work, discrete nanopillars arranged in a hexagonal lattice with a pitch of 300 nm were employed. Owing to the influence of insufficient phase sampling, a FOV of about 100° × 100° was allowed and the simulated focusing efficiency was about 14%.

When the quadratic metalens directly faces the object, the effective aperture can reach the maximum but at the cost of large spherical aberration, resulting in strong background noise and low imaging contrast, as discussed in Section 2.2. Using the configuration shown in Figure 2C, a near-infrared large-FOV metalens for outdoor imaging applications was proposed by Engelberg et al. in 2020 [88], as shown in the inset of Figure 8D. Even under the illumination of natural sunlight, good imaging quality was obtained over a FOV of ±15° with a band-pass filter (800 nm ± 5 nm) incorporated in front of the aperture stop. However, owing to the limitation of traditional discrete metasurfaces, the maximum diffraction efficiency was measured by about 20%, and the angular sensitivity makes the brightness of the center significantly higher than that of the outside, as shown in Figure 8D. As discussed in Section 2.2, such a configuration can only support a limited diffraction-limited FOV. In 2021, using the configuration shown in Figure 2D, Zhang et al. experimentally demonstrated a record diffraction-limited FOV of 178° × 178° by employing a 5 mm thick silica plate [82]. The employed wide-angle metalens has a diameter of 12 mm, a focal length of 4.48 mm, and a working wavelength of 940 nm. Furthermore, owing to the high efficiency and strong angular robustness of optimized streamline structures, the overall image brightness is uniform under the illumination of a normal near-infrared LED source, as can be seen intuitively from the right panel of Figure 5E. Besides nocturnal active imaging, this metacamera has also been demonstrated for day-time passive imaging, because a band-pass filter (940 nm ± 5 nm) had been incorporated in front of the sensor, thus showing great potential for night vision, spectral imaging, 3D imaging, etc.

### 4.2 Wide-angle Fourier transformation and beam steering

Fourier optics has been applied for many important applications, such as holography [29, 35, 137], spatial filtering [138], and compressed sensing [139]. As the basic optical element of Fourier optics, the Fourier lens can perform a Fourier transformation, and thus the spatial frequency of incident light can be measured at the focal plane. However, the operation of traditional Fourier lenses based on both refractive optics and diffractive optics usually is limited to the paraxial approximation and thus high-frequency information will be lost.

**Figure 9:**
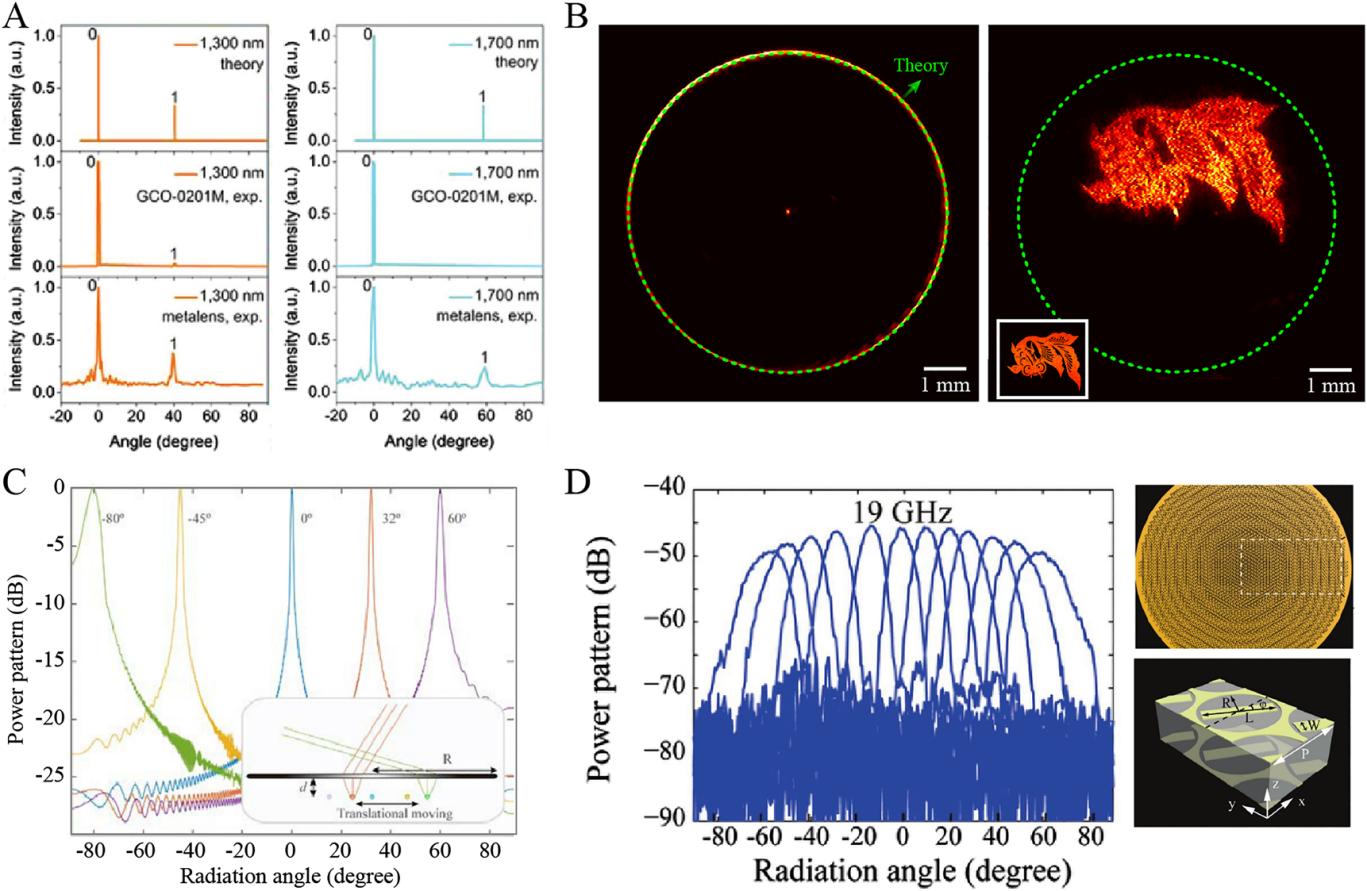
Applications of wide-angle metalenses for flat large-FOV imaging and beam steering. (A) Comparison of the 0th and 1st order diffraction at different wavelengths between the theoretical calculation and the corresponding measurement by Fourier transformation through GCO-0201M and the 1D quadratic metalens. Adapted with permission from ref. [89]. Copyright 2018, WILEY-VCH. (B) Spatial frequency measurement for a Bessel metalens (left) and meta-hologram (right) at 10.6 μm wavelength using a 2D quadratic metalens. Adapted with permission from ref. [82]. Copyright 2021, WILEY-VCH. (C) Schematic and performance of beam steering by the quadratic metalens operating at the wavelength of 532 nm. Adapted with permission from ref. [74]. Copyright 2017, Optical Society of America. (D) Measured far-field power patterns at the frequency of 19 GHz when a circularly polarized antenna is transversely shifted. Right insets show a perspective view of the quadratic metalens and the schematic of the unit cell. Adapted with permission from ref. [91]. Copyright 2018, WILEY-VCH.

Quadratic metalens provides a powerful framework for realizing optical Fourier transformations in a large incident angle range, because a focus offset of −*f*·sin*θ*_i_, which is required for a Fourier lens, can be perfectly realized. For example, wide-angle 1D Fourier transformation with a FOV of 120° was experimentally demonstrated by a 1D quadratic metalens composed of an array of dielectric waveguides [89]. When the incident linear polarization is parallel to the waveguide, the 1D quadratic metalens shows good performance within a broad bandwidth from 1100 to 1700 nm, and the focusing efficiency was in a range from 30% to 60% for different incident angles and operating wavelengths. The authors carried out a comparative experiment using the 1D quadratic metalens and a commercial Fourier lens (GCO-0201M). A diffraction grating with a period of 2 μm was employed as the target. As shown in Figure 9A, experimental results showed that the commercial Fourier lens could not work for incident angles larger than 30°. Because the employed 1D waveguides are sensitive to the incident polarization and suffer from insufficient phase sampling, it is hard to construct high-performance 2D Fourier devices for a larger FOV, as discussed in Section 3.2.

The quadratic metalens made of optimized streamline structures can address this issue well. Wide-angle 2D Fourier transformation with a FOV of 178° × 178° was experimentally demonstrated within the wavelengths from 9.3 to 10.6 μm [82]. The spatial frequency distributions of a Bessel metalens and a meta-hologram were measured using this 2D quadratic metalens with a diameter of 12 mm and a focal length of 5.12 mm. The blue dashed curves shown in Figure 9B indicate the theoretic results with the wave vector of *k*_0_sin45°. The measured results show good agreement with the theoretical results. The streamline quadratic metalens is very suitable for characterizations of PB-based meta-holograms in the laboratory, because it has a chiral response to prevent the detectors from damage by the focused co-polarized CP light (the 0th-order light). It means that such a single-chip metalens can replace a bulky combination of a quarter waveplate, a linear polarizer, and a Fourier lens, and thus more high-frequency information could be collected.

According to the principle the reciprocity, the quadratic metalens can be utilized to realize beam steering, providing a promising candidate for lightweight and wide-angle lidar systems. By adjusting the horizontal position and the distance between a point source and the quadratic metalens, the radiation direction and side lobe of the output beam can be flexibly controlled. This initial idea was proposed by Pu et al. in 2017 [74]. A large radiation angle range can be readily realized by horizontally moving the point source perpendicular to the optical axis with a distance of *s*, corresponding to a radiation angle of −sin^−1^(*f*/*s*). Figure 9C shows simulated results with the side lobe of radiation patterns below −20 dB, assuming *λ* = 532 nm, *R* = 100 μm, *f* = 50 μm, and *d* = 50 μm. In particular, the side lobe approximately increased by 8 dB after decreasing the distance between the point source and metalens from 50 μm to 46 μm. After one year, the wide-angle beam steering ability beyond 120° × 120° was experimentally demonstrated by Y. Guo et al. in the microwave band with the sidelobe below −20 dB [91], as shown in Figure 9D. The proposed ultrathin (≈0.127 *λ*) quadratic metalens was composed of bilayer geometric metasurfaces, where the transversal catenary field was utilized for high polarization conversion efficiency larger than 80%, while the wide-angle operation was ensured by the longitudinal catenary field and symmetry transformation. Owing to the insufficient phase sampling caused by discrete building blocks, it is hard to enable a larger FOV with high efficiency. The aforementioned quadratic metalens made of optimized streamline structures was also employed to realize laser scanning with a FOV larger than 170° × 170° at the wavelength of 10.6 μm [82]. Owing to its high diffraction efficiency and strong angular robustness, there is almost no noise or stray light for output beams, which is very important for the application of lidar techniques. For the further development of laser scanning techniques based on the quadratic metalens, one can combine one quadratic metalens with an array of vertical cavity surface lasers to achieve multi-channel laser parallel scanning, and multi-channel independent detection can be realized by the combination of another quadratic metalens and an array of avalanche photodiodes. Through the above configuration, all-solid-state, large-FOV, and high-frequency lidar systems become possible.

### 4.3 Simultaneous detection of spin and orbital angular momenta of light

All of the above quadratic metalenses have a radial-quadratic phase distribution, which enables optical symmetry transformation that translates the rotational symmetry of illumination to the translational symmetry of focus shift. Recently, the azimuthal-quadratic metalens has been shown to allow photonic momentum transformation [140]. Different from radial-quadratic metalenses mentioned above, the phase distribution of azimuthal-quadratic metalenses has a form of *l*_0_ *φ* ^2^/2, where *l*_0_ is the quadratic phase coefficient and *φ* is the azimuthal angle defined as tan^−1^(*y*/*x*). When incident light carrying orbital angular momentum with a topological charge of *l* passes through such an azimuthal-quadratic metalens, the phase carried by outgoing light can be given as:

(7) $$\phi \left(\varphi \right)=\frac{l_{0}}{2}\varphi^{2}+l\varphi =\frac{l_{0}}{2}{\left(\varphi +\frac{l}{l_{0}}\right)}^{2}-\frac{l^{2}}{2l_{0}}=\phi \left(\varphi +\frac{l}{l_{0}}\right)-\frac{l^{2}}{2l_{0}}$$

**Figure 10:**
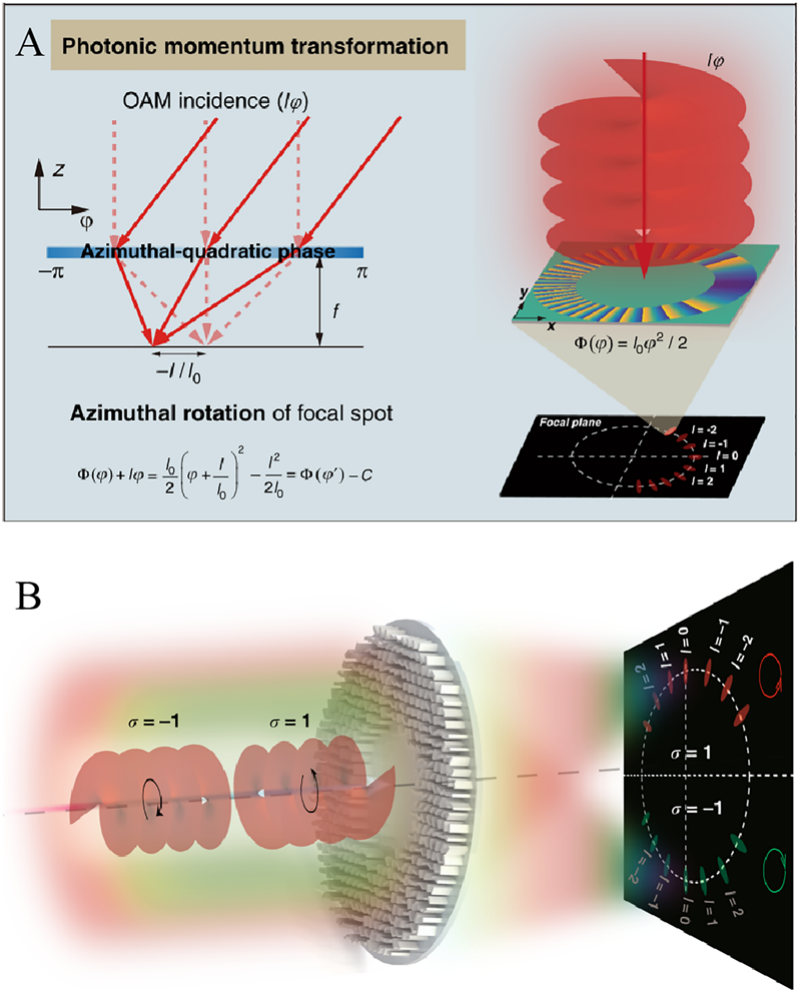
Applications of azimuthal-quadratic metalenses for detection of photonic angular momenta. (A) Principle of the photonic momentum transformation. (B) Schematic diagram of a spin-decoupled metasurface merging the geometric phase and propagation phase for simultaneous detection of spin and orbital angular momenta. Adapted with permission from ref. [140]. Copyright 2021, the authors. Distributed under the Creative Commons Attribution 4.0 International License (CC BY).

After neglecting the azimuth-independent term on the right side of the equation, one can conclude that the orbital angular momentum of light is transformed into the azimuthal rotation of the focal spot, as shown in Figure 10A. Therefore, the topological charge of incident light can be obtained after recording the azimuthal coordinate *φ*_*l*_ of the focal spot, with the value determined by –*l*_0_ *φ*_*l*_, where *φ*_*l*_ belongs to [−*π*, *π*]. In addition, spin-decoupled metasurface design merging the geometric phase and propagation phase was further employed to transform vortex beams of different spins into focusing patterns on two separated halves of the screen on a transverse focal plane. As a result, spin and orbital angular momenta of light can be simultaneously detected via the concept of momentum transformation. As a proof-of-concept demonstration, Guo et al. designed, fabricated, and characterized an azimuthal-quadratic metalens operating at the wavelength of 532 nm. As can be seen from Figure 10B, vortex beams with different topological charges and spins can be mapped into focusing patterns on different halves of the screen with different azimuthal angles.

## 5 Summary and outlook

In conclusion, wide-angle metalenses have attracted increasing attention in recent years, owing to their wide application prospect in surveillance, unmanned vehicles, onboard planes or satellites, medical science, and many others [82], [83], [84], [85], [86], [87], [88], [89], [90], [91], [92], [93], [94]. Compared with the traditional one, metasurface design has advantages of thin thickness, flat geometry, light weight, and so on. Five basic configurations for sing-chip wide-angle metalenses are summarized in this review. It is concluded that all wide-angle metalenses have a large phase gradient at the edge and require strong angular robustness for their basic building blocks. These properties make traditional metasurface designs hard to construct high-performance wide-angle metalenses. On the one hand, according to the diffraction theory, discrete metasurfaces suffer from insufficient phase sampling, because a small unit pitch will cause unpredicted electromagnetic coupling, resulting in strong noise and low efficiency. On the other hand, the traditional design method utilizes the phase response of periodic unit elements as the reference standard for device design. However, the assumption of periodic boundary conditions can be approximately established only when the phase gradient is small, which is contrary to the large phase gradient characteristic of wide-angle metalenses. As a result, the real electromagnetic responses including phase, amplitude, and polarization deviate from the ideal one.

This review introduces two potential methods, namely adjoin optimization and catenary optics, to overcome these limitations. The advantages and disadvantages of adjoint-based topology optimization and adjoint-based shape optimization are discussed. It is concluded that the former has higher design freedom, while the latter is more suitable for parameterized devices, showing more potential to realize the optimal design of large-area devices. Since adjoint optimization requires time-consuming full-model simulations, how to optimize large-area devices is still a big challenge. We think the combination of deep learning and adjoint-based shape optimization may be a good solution. In addition, one successful none-time-consuming solution, namely isophase streamline optimization, is proposed to construct high-efficiency, broadband, and wide-angle catenary-like metasurfaces. It is commonly regarded that catenary-like structures outperform discrete structures [127], because they can enable continuous wavefront control, thus avoiding insufficient phase sampling. Through the isophase streamline optimization [82], the parasitic propagation phase in the dielectric streamline structures is suppressed, so pure and precise geometric phase control results in high diffraction efficiency in a wide spectral and angular range, which is very suitable for the construction of wide-angle metalenses.

Following the mechanisms, challenges, and design methods of wide-angle metalenses, representative functional devices and applications including wide-angle imaging, Fourier transformation, beam steering, and photonic momentum detection are reviewed. In particular, a record diffraction-limited FOV of 178° × 178° was demonstrated for wide-angle imaging by quadratic metalenses incorporated with an aperture stop, and a FOV larger than 170° × 170° was realized for laser scanning [82]. We believe that these metalenses will soon be practical in many fields, such as night vision, spectral imaging, 3D imaging, lidar detection, etc., in which lasers or narrowband light sources are employed. For wider applications or research, however, there are still many challenges to be addressed.

First of all, how to improve spectral bandwidth is an urgent challenge. Although geometric-phase-based metalenses can operate within a wide spectral band and have considerable efficiencies, the chromatic aberration still exists and the imaging quality decreases rapidly with the increase of light source bandwidth, especially for the off-axis FOV. Broadband achromatic metalenses have been demonstrated through dispersion engineering of subwavelength structures, but up to now, the reported ones have a very small aperture or NA [65, 72, 78]. Fortunately, multiwavelength achromatic metalenses get rid of these restrictions and show potential for wide-angle holographic display, virtual reality display, and augmented reality display [103, 104, 141], [142], [143]. How to design large-scale broadband achromatic wide-angle metalenses is still an open question. The combination of traditional lenses with positive dispersion and metalenses with negative dispersion should be a feasible compromise to improve achromatic bandwidth [144, 145].

Secondly, further study is required for breaking the trade-off of wide-angle metalenses between the FOV and effective NA. In most designs, the yielded phase profile is independent of the incident angle [89, 90, 95], which is the fundamental limitation of the aforementioned trade-off. Early study has shown that near-ideal wide-angle metalenses can be realized at several discrete FOVs when employing the angular dispersion of subwavelength structures [90]. As a result, it is expected that the effective NA could be further increased through the joint design of ray tracing and numerical simulation, where angular dispersion is considered and artificially engineered.

Thirdly, it should be interesting if the FOV of metalenses can break through 180°. For planar configurations, diffraction metagratings or metaholograms can easily operate in both transmission mode and reflection mode [16, 146], [147], [148], but how to control the transmission field and reflection field independently and arbitrarily still requires further research. One potential approach is to utilize the local polarization-selective interference effect that is enabled by merging the geometric phase and propagation phase [16, 146]. For imaging applications, one should probably consider combining traditional lenses and a wide-angle metalens to make FOV exceed 180°. Specifically, the curved optical component might be necessary to compress light rays from the back of the metalens to its front side, and then the light tracing method can be employed to optimize their curvatures, materials, relative positions, and phase distributions.
